# Impact of baseline impedance of pulmonary vein antrum on success of catheter ablation for paroxysmal atrial fibrillation guided by ablation index

**DOI:** 10.1186/s12872-022-02530-y

**Published:** 2022-04-19

**Authors:** Yuanjun Sun, Xianjie Xiao, Xiaomeng Yin, Lianjun Gao, Xiaohong Yu, Rongfeng Zhang, Zhongzhen Wang, Shiyu Dai, Yanzong Yang, Yunlong Xia

**Affiliations:** Department of Cardiology, The First Affiliated Hospital of Dalian Medical University, 222 Zhongshan Rd, Dalian, 116011 Liaoning China

**Keywords:** Atrial fibrillation, Ablation index, Baseline impedance, Ablation, Pulmonary vein antrum

## Abstract

**Objective:**

Ablation index (AI) is an effective ablation quality marker. Impedance is also an important factor for lesion formation. The present study evaluated the influence of the baseline impedance in the effect of ablation for atrial fibrillation (AF) guided by AI.

**Methods:**

This was a retrospective study. 101 patients with paroxysmal AF (PAF) were enrolled. All patients underwent radiofrequency ablation guided by the same AI strategy. The ablation strategy was pulmonary vein (PV) isolation with non-PV triggers ablation. The baseline impedance of the ablation points was recorded. The patients were followed up every 3 months or so.

**Results:**

During a median follow-up of 12 (4–14) months, freedom from AF/atrial tachycardia recurrence were 82.2%. No difference existed in baseline characteristics between the success group and the recurrence group. The average baseline impedance was 124.3 ± 9.7 Ω. The baseline impedance of the ablation points in success group was lower compared to the recurrence group (122.9 ± 9.4 vs. 130.5 ± 8.8 Ω, *P* < 0.01). The ratio of impedance drop in the success group was higher than the recurrence group ([8.8 ± 1.4]% vs. [8.1 ± 1.2]%, *P* = 0.03). Multivariate analysis revealed that baseline impedance, PAF duration and AI were the independent predictors of AF recurrence. The cumulative free of recurrence rate of low-impedance group (≤ 124 Ω, n = 54) was higher than that of high-impedance group.

**Conclusion:**

Baseline impedance correlates with clinical outcome of radiofrequency ablation for PAF guided by AI. Higher impedance in the same AI strategy may result in an ineffective lesion which probably causes recurrence.

**Supplementary Information:**

The online version contains supplementary material available at 10.1186/s12872-022-02530-y.

## Introduction

Pulmonary vein isolation (PVI) is the cornerstone of catheter ablation for atrial fibrillation (AF) [1, 2]. Avoiding gaps of the PVI circle is of great importance for effect of ablation. Achieving durable PVI is necessary for the efficacy of ablation of paroxysmal atrial fibrillation (PAF). Recurrences are mainly due to pulmonary vein (PV) reconduction in PAF because of the insufficient lesion formation caused by ablation. Lesion formation caused by radiofrequency (RF) current depends on several parameters, such as the power, duration of ablation, contract force (CF), current [3, 4].

Ablation index (AI) is an integral formula including contact force, power, and duration, which is an effective predictor for lesion formation. AI has been widely used for guiding PVI. However, the index algorithm does not have the impedance parameter, which may also influence the current and ablation thermal effect. Different baseline impedance in the same AI may cause different lesion. In the present study, we aimed to access the impact of baseline impedance of PV antrum on effect of catheter ablation for PAF guided by AI.

## Methods

### Patient population

This was a retrospective study. 101 patients with drug-refractory symptomatic paroxysmal AF (PAF) experienced first catheter ablation in our center from November 2017 to December 2018 were enrolled. All patients involved were free of congenital heart diseases, thyroid dysfunction, moderate-to-severe valvular heart disease or prior cardiac surgery. All patients had signed the consent form of operation before the procedure. The data are anonymous, and no additional informed consent of analyzing data was required.

### Catheter ablation protocol

Left atrial thrombus was excluded through cardiac computer tomography scan or transesophageal echocardiography for each individual before procedure. The surface return patch was positioned on the left part of the waist. Ablation procedure was performed in local anesthesia with dezocine for analgesia. Under fluoroscopic guidance (Innova 2000, GE, WI, USA), one or two multipolar catheters (MicroPort, Shanghai, China) were placed in the coronary sinus and right ventricle (if necessary) through the right femoral vein (just one catheter in coronary sinus) or left femoral vein (two catheters in coronary sinus and right ventricle). Two transseptal sheaths (Synaptic Medical, Beijing, China) were introduced into the right femoral vein. After double transseptal punctures were performed, anticoagulation was started by a bolus administration of 100 IU/kg heparin followed by continuous intravenous heparin infusion to maintain an activated clotting time of 300–350 s. A CF catheter (SmartTouch, Biosense Webster, Diamond Bar, USA) and a multispline (PentaRay, Biosense Webster, Diamond Bar, USA) or circular (MicroPort, Shanghai, China) mapping catheter were inserted to left atrium (LA) through the two transseptal sheaths.

Electroanatomic 3-dimensional mapping of the LA and PV ostia was performed by the Carto3 system (Biosense Webster, Diamond Bar, USA). Identification of the LA-PV junction was based on the anatomy, CF vector and local potential characteristics. Then point-by-point RF delivery was performed on the LA-PV junction to encircle the ipsilateral PVs to achieve isolation. The distance between the neighboring points was less than 6 mm. The saline irrigation flow was 2 mL/min during catheter manipulation, 17 mL/min during RF ablation. RF delivery was performed at a constant power of 35 W or 30 W (when at posterior left inferior PV). Ablation was guided by AI target values for each lesion as follows: 500–520 for anterior/roof segments and 350–400 for posterior/inferior segments of the LA. If a trigger (premature atrial contraction) arose from superior vena cava (SVC) after bilateral PVI, segmental isolation of SVC was required. After PVI and SVC isolation (if necessary), 30 min was taken for monitoring the bidirectional block. If conduction recovery occurred, re-ablation was performed for isolation. 101 operations were performed by one main doctor who operated the ablation catheter.

### Impedance parameters analysis

Baseline impedance was recorded around the right and left pair of PVs at the ablation site. The average impedance of the bilateral PVs ablation points was calculated. The average impedance decrease of the ablation sites around the PVs was also calculated.

### Post-procedural management and follow-up

Patients received anticoagulation therapy with dabigatran (110 mg twice daily), rivaroxaban (20 mg once daily) or warfarin with target INR of 2–3 for at least 3 months after the procedure. The patients were followed up in our center at regularly scheduled visits every 3 months or so. During the regular follow-up, a 24-h Holter was obtained. When the patients experienced palpitation, ECG and Holter were also performed to assess for arrhythmia recurrence. Success was defined as being free of any atrial tachyarrhythmia lasting longer than 30 s after the 3-month blanking period.

### Statistical analysis

Continuous variables were summarized as mean ± standard deviation. Categorical variables were summarized by frequency and percentage tabulations. Follow-up period was presented as a median with the entire range. Comparisons between groups were performed with either the Student’s unpaired t-test or Wilcoxon rank-sum test or Chi-square test. Normal distribution was verified by Kolmogorov–Smirnov test. Cox's proportional hazards model was used to assess the independent predictors of the recurrence by backward Likelihood Ratio (LR) method. The cumulative freedom from recurrence after ablation was evaluated using Kaplan–Meier event-free survival analysis. All statistical tests were 2 tailed and performed using the SPSS 19.0. A *P* value < 0.05 was considered statistically significant.

## Results

### Patient characteristics

All patients (age 61.7 ± 8.5 years, 58.4% males) completed the study protocol. The time course of AF was 3.2 ± 3.1 years. The mean left ventricular ejection fraction (LVEF) was (58.9 ± 2.4)%, and left atrial size was 38.2 ± 4.6 mm (Additional file 1).

### Procedural characteristics and follow-up

**Fig. 1:**
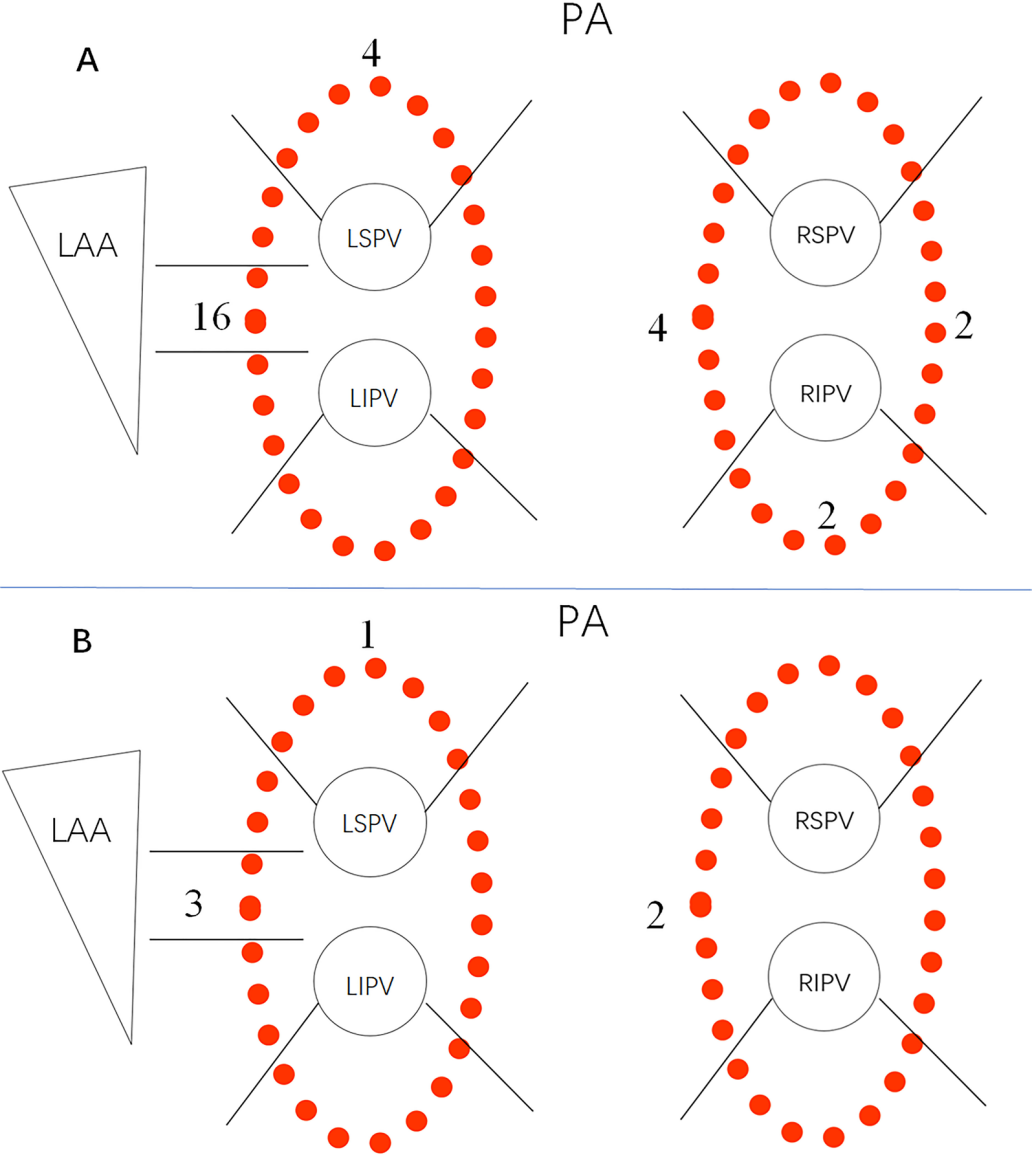
The locations of the gaps after first-pass ablation (**A**) and gaps of acute reconnection (**B**)

**Fig. 2:**
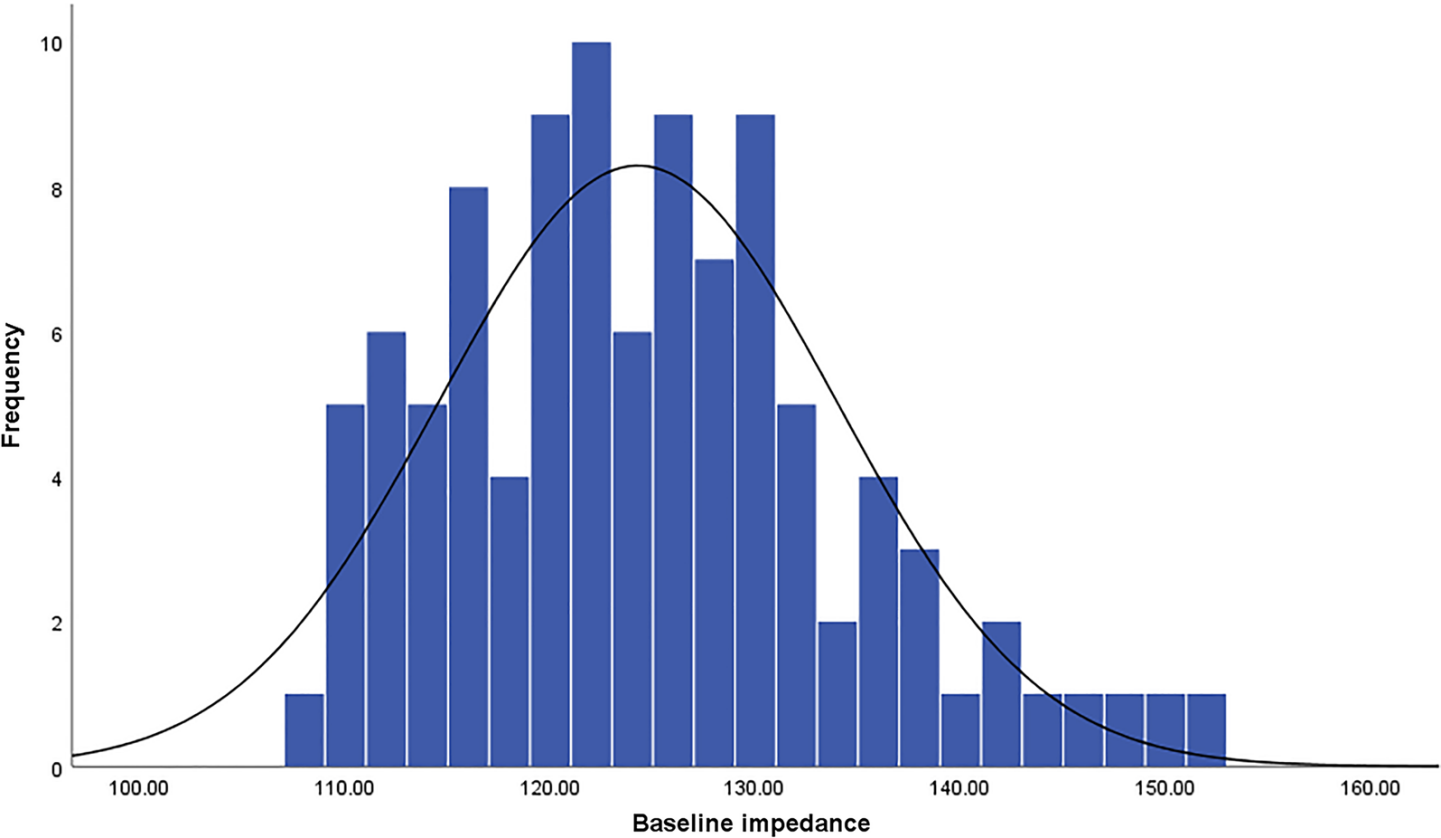
The average baseline impedance was 124.3 ± 9.7 Ω (n = 101, ranged from 108 to 151 Ω)

All PVs were successfully isolated in all the patients. The first-pass PVI rate was 88.6% (179/202). Bilateral PVIs were not achieved 2 patients and unilateral PVI was not achieved in 19 patients. 28 gaps existed in the 23 ablation circles (Fig. 1). Acute reconnection was observed in 5 patients in the 30 min for monitoring. Bilateral PVs’ conduction recovered in 1 of the 5 patients. The gaps were distributed as the Fig. 1 shows. 3 patients experienced segmental SVC isolation. None SCV reconnection occurred within the monitoring time. The baseline impedance was subject to normal distribution (*P* = 0.20) as the Fig. 2 shows. The average baseline impedance was 124.3 ± 9.7 Ω (ranged from 108 to 151 Ω). No patient experienced pericardial effusion during ablation.

During a median follow-up of 12 (4–14) months after the procedure, 83 patients (82.2%) were free of recurrence without antiarrhythmics. No difference existed in characteristics except baseline impedance between the success group and the recurrence group (Table 1). The first-pass PVI rate was higher in success group (91.0 vs. 77.8%, *P* = 0.03). The average baseline impedance of the ablation points in success group was lower compared to the recurrence group (122.9 ± 9.4 vs. 130.5 ± 8.8 Ω, *P* < 0.01). No difference existed in the impedance drop between the two groups. However, the ratio of impedance drop in the success group was higher than the recurrence group ([8.8 ± 1.4]% vs. [8.1 ± 1.2]%, *P* = 0.03) (Table 1). Predictors of the outcome of ablation (age, left atrial diameter, PAF duration, body mass index (BMI), LVEF, First-pass PVI rate, AI, baseline impedance, ratio of impedance drop) were evaluated by a Cox proportional hazards model. A Cox regression multivariate analysis revealed that baseline impedance (HR 1.06, 95% CI 1.01–1.10, *P* < 0.01), PAF duration (HR 1.18, 95% CI 1.06–1.32, *P* < 0.01) and AI (HR 0.92, 95% CI 0.86–0.99, *P* = 0.03) were the independent predictors of AF recurrence (Table 2).

**Table 1:** Patient characteristics between the success group and recurrence group

	Success group n = 83	Recurrence group N = 18	*P* value
Age, years	61.4 ± 8.5	63.2 ± 8.5	0.40
Male (%)	52 (62.7)	7 (38.9)	0.06
PAF Duration, years	2.9 ± 2.5	4.7 ± 4.7	0.13
BMI (kg/m2)	25.1 ± 2.6	24.8 ± 3.0	0.70
CHA_2_DS_2_-VASc score	1.2 ± 0.9	1.8 ± 1.3	0.07
LVEF (%)	58.0 ± 2.5	57.8 ± 2.0	0.76
Left atrial diameter, mm	38.0 ± 4.7	38.9 ± 4.4	0.45
Hypertension, n (%)	18 (21.7%)	4 (22.2%)	0.96
Coronary disease, n (%)	6 (7.2%)	3 (16.7%)	0.414
Diabetes mellitus, n (%)	6 (7.2%)	3 (16.7%)	0.20
AI	421.4 ± 7.6	417.6 ± 6.7	0.06
Baseline impedance (Ω)	122.9 ± 9.4	130.6 ± 8.9	< 0.01
Impedance drop (Ω)	10.8 ± 1.4	10.5 ± 1.3	0.41
Ratio of impedance drop (%)	8.8 ± 1.4	8.1 ± 1.2	0.03
First-pass PVI, n (%)	151 (91.0)	28 (77.8)	0.03
Number of ablation points	74.1 ± 7.7	76.4 ± 6.7	0.24

**Table 2:** Cox regression multivariate analysis to assess predictors of recurrence

Factor	Hazard ratio (95%CI)	*P* value
Age (years)*	1.01 (0.95–1.09)	0.66
LAD (mm)*	1.02 (0.92–1.14)	0.71
BMI (kg/m^2^)*	0.92 (0.77–1.11)	0.40
LVEF (%)*	0.95 (0.75–1.19)	0.64
Ratio of impedance drop (%)*	0.85 (0.53–1.35)	0.48
First-pass PVI rate (%)*	0.45 (0.18–1.10)	0.08
Baseline impedance (Ω)^#^	1.06 (1.01–1.10)	< 0.01
PAF duration (years)^#^	1.18 (1.06–1.32)	< 0.01
AI^#^	0.92 (0.86–0.99)	0.03

*The HRs and *P* values of the variables in the equation at the first step of the backward LR method in Cox’s proportional hazards model

^#^The variables in the equation at the last step of the backward LR method

### Impedance of the patients with gaps

The average baseline impedance of the 21 patients with gaps after first-pass ablation has no statistical difference with the impedance (127.0 ± 9.4 vs. 123.5 ± 9.7Ω, *P* = 0.14) of the patients with first-pass isolation.

### Low-impedance group and high-impedance group

**Fig. 3:**
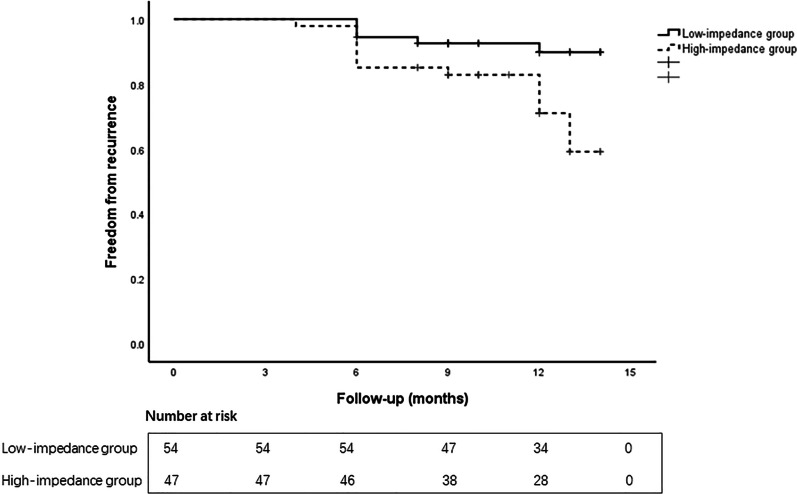
Freedom from recurrence of the low-impedance group and high-impedance group. Kaplan–Meier curve showed that cumulative freedom from recurrence was higher in low-impedance group than that in high-impedance group (log-rank test, *P* = 0.02)

According to the mean baseline impedance (124 Ω) over all procedures, the patients were split into 2 groups: the low-impedance group (≤ 124 Ω, n = 54) and the high-impedance group (> 124 Ω, n = 57). There was no statistical difference in the age, time course of AF, left atrial size, LVEF, BMI, CHA_2_DS_2_-VASc score, and ratios of gender, hypertension, coronary heart disease and diabetes mellitus between the two groups (Table 3). On the aspect of parameters of the ablation, no statistical difference was found in AI and impedance drop (Table 4). First-pass isolation was obtained in 98/108 circles (90.7%) in low-impedance group, while was achieved in 86/94 circles (86.2%) in high-impedance group without significant difference. The number of patients without recurrence were 49 patients (90.7%) and 34 patients (72.3%) in the low-impedance group and high-impedance group, respectively (*P* < 0.05). And cumulative free of recurrence rate of low-impedance group was higher than that of high-impedance group as the Kaplan–Meier curve showed (Fig. 3).

**Table 3:** Patient characteristics between the low-impedance group and high-impedance group

	Low-impedance group (n = 54)	High-impedance group (n = 47)	*P* value
Age (years)	60.6 ± 9.9	63.0 ± 6.5	0.14
Male (%)	33 (61.1)	26 (55.3)	0.56
PAF duration (years)	2.7 ± 2.6	3.7 ± 3.5	0.11
BMI	24.8 ± 2.5	25.3 ± 2.8	0.65
CHA_2_DS_2_-VASc score	1.3 ± 1.1	1.4 ± 1.0	0.78
LVEF (%)	57.9	58.0	0.82
Left atrial diameter, mm	38.4 ± 4.1	37.9 ± 5.2	0.61
Hypertension, n (%)	15 (27.8)	7 (14.9)	0.12
Coronary disease, n (%)	4 (7.4)	5 (12.8)	0.83
Diabetes mellitus, n (%)	4 (7.4)	5 (10.6)	0.83

**Table 4:** Parameters of the ablation between the low-impedance group and high-impedance group

	Low-impedance group (n = 54)	High-impedance group (n = 47)	*P* value
AI	419.7 ± 7.6	421.8 ± 7.5	0.18
Baseline impedance (Ω)	117.0 ± 4.5	132.5 ± 6.9	< 0.01
Impedance drop (Ω)	11.0 ± 1.5	10.4 ± 1.3	0.05
Ratio of impedance drop (%)	9.4 ± 1.4	7.9 ± 1.0	< 0.01
First-pass PVI rate, n (%)	98 (90.7)	81 (86.2)	0.28

## Discussion

### Main findings

This study explored the relationship between baseline impedance and success of PAF ablation. We demonstrated the baseline impedance might influence success rate of PAF ablation. It is the first clinical study that assessed the effect of PAF RF ablation guided by AI in different baseline impedance. Different baseline impedance in the same AI strategy may cause different ablation effect. Higher impedance may reduce the thermal injury of RF which may lead to higher recurrence of AF ablation.

### Recurrence and reconnection of PV

Transmural lesion is necessary to achieve sustained PVI in AF ablation. Durable PVI is associated with a lower risk of AF recurrence after catheter ablation. PV reconnection is the most common electrophysiological factor in patients with atrial fibrillation recurrence [5–8].Acute isolation of the PV may not suggest the thermal injury of the tissue was enough. In the study, all patients achieved the PVI and the first-pass isolation rates were also similar between the low impedance group and high impedance group. However, the recurrence rates were different between the two groups. Although we did not identify the mechanism of recurrence, the main factor may be PV reconnection. We found the phenomenon that baseline impedance might influence the success rate of ablation in PAF guided by AI. The recurrence was related to baseline impedance, which might result in a more frequent PV reconnection after procedure.

### AI and ablation lesion

Acute electrical PVI is not equal to the durable transmural lesion. Reversible tissue injury can resolve along with the time after procedure, gaps occurs and PV reconnection can induce AF recurrence of atrial tachycardia [9].The quality of RF ablation at each ablation point is associated with electrode-tissue CF, delivered power, ablation duration, which AI that incorporates the preceding parameters in an integral formula showed as $AI={\left(k\times {\int }_{0}^{t}C{F}^{a}\left(t\right){P}^{b}\left(t\right)dt\right)}^{c}$ (CF is contact force, *P* is RF power, T is ablation time, and a, b, c, and k are constants) [10]. AI guided ablation is associated with significant improvements in acute PV reconnection and recurrence rate compared to just CF guided ablation [11]. AI, as a marker of lesion quality, however does include the impedance. In the present study, all patients experienced ablation guided by same AI strategy, but the recurrence rate were different between the low-impedance and high impedance. Maybe the impedance influenced the ablation effect. Different baseline impedance may require different AI aim.

### Baseline impedance and ablation lesion

The study revealed that high baseline impedance was associated with a higher rate of AF recurrence following RF ablation at a median 12-month follow-up. This might be due to inefficient RF energy delivered and lesion formation.

RF current generates thermal injury of the tissue when it flows through the myocardium. The degree of thermal injury of RF to the myocardium is proportional to the current density at the tip, which is related to the impedance [12]. The scope of the lesion is determined by the amount of current delivered [13]. The lesion sizes were significantly larger with lower impedance during irrigated ablation and the size was smaller in high impedance ablations at similar power settings [14]. Lesion formation is related to current. A study by Barkagan et al. [15] suggested that baseline impedance had negative correlation with current squared. According to in vitro and in vivo experiments on swine models, ablation with fixed power resulted in increased current and larger lesion sizes at location with lower baseline impedance. Also, Bourier et al. [16] verified that sizes of ablation lesions were significantly different with same power but different impedances in porcine in vitro models. And current changed with wide range with variations in impedance in clinical RF ablation. According to the Ohm’s law equation, P = I^2^R (P = power, I = current, R = resistance/impedance), when the impedance of the circuit increases, the current output decreases and vice versa. So higher impedance corresponds to lower current and lower current density that can result in lower delivered energy to the myocardium and smaller lesion formation. The delivered energy could be variation in individuals because of the different baseline impedance. Impedance may act as an influential factor in RF ablation.

In the present study, the average baseline impedance of the 21 patients with gaps after first-pass ablation has no statistical difference with the impedance of the patients without gaps. Maybe the gaps were related to the stability of the catheter and the thickness of the local tissue, because most gaps located in the ridge of the left atrial appendage and the carinas of the PVs. The ridge and carinas made it more difficult to stabilize the ablation catheter when ablating and gaps occurred more frequently than other areas.

### Influence factors of impedance

Impedance represents the resistance to current flow that related to the local myocardium, RF generator, ablation catheter, return electrode patch distance to the heart, and the quality of skin contact and cabling [17–20]. The range of baseline impedance observed during human ablations was between 100 and 120 Ω or above [14, 21]. In this study, the average baseline impedance was 124.3 ± 9.7 Ω, which probably was associated with kinds of factors. The number and location of the return patches of the RF generators can influence the baseline impedance. Baseline impedance was associated with the position of the surface return patch. Increasing the number of patches or decreasing their distance to the heart can reduce the baseline impedance and increasing the effect of RF ablation [22]. Shapira-Daniels et al. reported that ablation after reducing the baseline impedance was a simple, safe, and effective technique for increasing the effect of ablation for ventricular arrhythmia refractory to regular ablation with irrigated catheters. At the same AI, a lower baseline impedance may produce larger current and more severe lesion during ablation. On the basis of the above mechanism and results of the present study, altering the position of the return patch to getting closer to the heart or adding return patches for patients with high baseline impedance may increase the effectiveness of ablation at same AI guidance strategy because of reduced impedance. Or in the patients with high baseline impedance, a higher target AI may be reasonable.

The complications of catheter ablations were higher in patients with low BMI [23, 24]. Maybe one reason is that it is because of the baseline impedance is lower accompanied with BMI. It is possible that the impedance is related to tissue between the myocardium and return patch, which adipose contributes greatly. In the study, the average BMI between the low-impedance and high-impedance groups showed no statistical different. Whereas the BMI showed a decreased trend in low-impedance group (24.8 ± 2.5 vs. 25.3 ± 2.8, *P* = 0.65), also the impedance drop displayed a similar increased trend (11.0 ± 1.5 vs. 10.5 ± 1.4, *P* = 0.08) which revealed a probably deeper lesion [25]. Maybe the body fat percent is more accurate than BMI, which needs further study.

### Study limitations

There are several limitations of the present study. The sample size was relatively small which needs larger studies for exploring more precise conclusion. The comparison of impedance between the area with and without gaps after first-pass ablation was not performed because of the too much difference in quantity. Maybe the gap was an area and not one ablation point, we could not know which point of the first-pass ablation was the accurate location of the gap. So we could not clarify the detailed parameters (like baseline impedance, AI, etc.) of the gaps. Also, it was unclear if the recurrence was due to PV reconnection or another mechanism, which could be assessed during a redo procedure. Modulating the baseline impedance or AI aim for patient with high impedance may improve the clinical outcome after procedure which we did not perform.

## Conclusions

Baseline impedance may influence the clinical outcome of RF ablation for PAF guided by AI. Higher impedance in the same AI strategy may result in an ineffective lesion which probably causes recurrence.

## Supplementary Information

**Additional file 1.** Raw data of the study.
